# Mycorrhizal-Assisted Phytoremediation and Intercropping Strategies Improved the Health of Contaminated Soil in a Peri-Urban Area

**DOI:** 10.3389/fpls.2021.693044

**Published:** 2021-07-02

**Authors:** María Teresa Gómez-Sagasti, Carlos Garbisu, Julen Urra, Fátima Míguez, Unai Artetxe, Antonio Hernández, Juan Vilela, Itziar Alkorta, José M. Becerril

**Affiliations:** ^1^Department of Plant Biology and Ecology, University of the Basque Country (UPV/EHU), Leioa, Spain; ^2^Department of Conservation of Natural Resources, NEIKER, Basque Research and Technology Alliance (BRTA), Derio, Spain; ^3^Centro de Estudios Ambientales, Vitoria-Gasteiz, Spain; ^4^Department of Biochemistry and Molecular Biology, University of the Basque Country (UPV/EHU), Leioa, Spain

**Keywords:** alfalfa, poplar, mycorrhizal inoculation, organic contaminants, plant diversity, soil microbial properties

## Abstract

Soils of abandoned and vacant lands in the periphery of cities are frequently subjected to illegal dumping and can undergo degradation processes such as depletion of organic matter and nutrients, reduced biodiversity, and the presence of contaminants, which may exert an intense abiotic stress on biological communities. Mycorrhizal-assisted phytoremediation and intercropping strategies are highly suitable options for remediation of these sites. A two-year field experiment was conducted at a peri-urban site contaminated with petroleum hydrocarbons and polychlorinated biphenyls, to assess the effects of plant growth (spontaneous plant species, *Medicago sativa*, and *Populus* × *canadensis*, alone *vs.* intercropped) and inoculation of a commercial arbuscular mycorrhizal and ectomycorrhizal inoculum. Contaminant degradation, plant performance, and biodiversity, as well as a variety of microbial indicators of soil health (microbial biomass, activity, and diversity parameters) were determined. The rhizosphere bacterial and fungal microbiomes were assessed by measuring the structural diversity and composition via amplicon sequencing. Establishment of spontaneous vegetation led to greater plant and soil microbial diversity. Intercropping enhanced the activity of soil enzymes involved in nutrient cycling. The mycorrhizal treatment was a key contributor to the establishment of intercropping with poplar and alfalfa. Inoculated and poplar-alfalfa intercropped soils had a higher microbial abundance than soils colonized by spontaneous vegetation. Our study provided evidence of the potential of mycorrhizal-assisted phytoremediation and intercropping strategies to improve soil health in degraded peri-urban areas.

## Introduction

Abandonment of agricultural and industrial sites in the periphery of many cities often results in the presence of randomly distributed vacant areas that are highly vulnerable and susceptible to uncontrolled deposition of wastes, thus contributing to soil degradation and contamination (Németh and Langhorst, 2014; Gómez-Sagasti et al., 2018; Míguez et al., 2020). Properly managed, these abandoned sites not only can be a valuable ecological resource, for instance by providing ecosystem services (Nassauer and Raskin, 2014; Herrmann et al., 2016) and harboring biodiversity (Delgado de la Flor et al., 2020; Perry et al., 2020), but can also afford an opportunity for land redevelopment and hence economic and social benefits (Kim et al., 2018). Implementation of sustainable, cost-efficient *in-situ* interventions, such as phytoremediation strategies, can improve soil health and thus contribute to the redevelopment of degraded peri-urban vacant lands (Míguez et al., 2020).

Phytoremediation is a gentle remediation option (Kidd et al., 2015) that uses plants and associated microbes to restore soils contaminated with inorganic and/or organic compounds. A variety of characteristics make phytoremediation an attractive alternative to conventional physicochemical methods of soil remediation: low capital and maintenance costs, easy start-up, non-invasiveness, environmentally friendly character, high public acceptance, etc. (Alkorta et al., 2010). The success of phytoremediation depends on the ability of certain plant species, together with their associated rhizosphere microorganisms, to stabilize (phytostabilization) and/or extract (phytoextraction) inorganic (metal) contaminants and/or degrade organic compounds (rhizoremediation). Thus, phytoremediation can gradually alleviate the potential environmental risks caused by contaminants and improve soil health (Vangronsveld et al., 2009).

Nitrogen-fixing herbaceous species such as alfalfa (*Medicago sativa* L.) and fast-growing trees such as poplar (*Populus* L.) are highly suitable candidates for phytoremediation initiatives. Alfalfa, an important perennial forage crop, has been widely used for phytoremediation of soils contaminated with metals (Bonfranceschi et al., 2009), petroleum hydrocarbons (Phillips et al., 2009; Panchenko et al., 2017), and aromatic hydrocarbons (Fan et al., 2008; Teng et al., 2011; Marchand et al., 2016). This leguminous plant species has many advantages for phytoremediation purposes, such as a deep root system favorable for the establishment of rhizosphere microorganisms (Kirk et al., 2005), high yield, and drought tolerance (Zhang M. et al., 2019). Furthermore, alfalfa requires little maintenance (it does not need annual plowing and sowing) and can fix up to 230 kg N ha^–1^ yr^–1^ (Tisdale and Nelson, 1974). Because of their high biomass production rates, poplars are increasingly chosen for reforestation of abandoned agricultural lands, revegetation of areas that have been degraded by industrial contamination (Tognetti et al., 2013; Foulon et al., 2016), and phytoremediation and phytomanagement strategies (Ciadamidaro et al., 2017). Many poplars are pioneer species, a trait that is inherently linked to their fast growth rate and deep root system (Hu et al., 2019). As with alfalfa, poplars have been extensively used for phytoremediation of metal- and hydrocarbon-contaminated soils (Lingua et al., 2008).

Although most phytoremediation studies have been conducted using a single plant species, the potential economic and environmental benefits of intercropping with trees and herbaceous species are well established (Taghiyari and Sisi, 2012). In particular, a poplar-alfalfa intercropping system has high potential for *in-situ* phytoremediation of contaminated soils.

The low availability of nutrients and contaminants in soil, as well as the unacceptably long time period required for effective remediation, are often the most important limitations of phytoremediation in the field. Two soil-management practices (used singly or in combination) that can assist phytoremediation processes are application of organic amendments (Gómez-Sagasti et al., 2018; Míguez et al., 2020) and inoculation of microorganisms such as mycorrhizal fungi, which can form symbiotic associations with most land plants (Miransari, 2011). Under natural conditions, *Populus* species and hybrids have the unique property of being colonized by both endo- and ectomycorrhizal (arbuscular) fungi, making them unique model systems for study of the interactions between plants and microorganisms (Martin et al., 2004; Karliński et al., 2010). In addition to improving plant mineral nutrition and health, mycorrhizal interactions are involved in protecting plants against soil-borne stresses (Szuba, 2015).

The objective of remediation processes must be not only to remove contaminants (and their associated risks) from the soil, but to recover soil health (Epelde et al., 2009; Gómez-Sagasti et al., 2012). Reliable and sensitive indicators of soil health are needed to properly assess the efficiency of phytoremediation processes. Besides the traditional physicochemical indicators, biological indicators of soil health (including plant, animal, and microbial parameters) are widely used to evaluate the effectiveness of phytoremediation processes (Burges et al., 2016; Lacalle et al., 2018; Anza et al., 2019).

This study investigated the benefits of two phytoremediation strategies for the physicochemical and biological properties of a contaminated peri-urban vacant soil: (i) intercropping of alfalfa with hybrid black poplar *vs.* monocultures of each species; and (ii) inoculation with a commercial mycorrhizal inoculum *vs.* non-inoculation. Treatment effects were evaluated in terms of the degradation of soil organic contaminants, plant performance (i.e., plant diversity, plant growth, and ecophysiological traits), and the status of soil microbial communities (i.e., microbial biomass, activity, and functional and structural diversity). We hypothesized that the selected treatments (mycorrhizal-assisted phytoremediation and intercropping) would enhance plant performance and improve the health of a contaminated soil in a peri-urban area.

## Materials and Methods

### Site Description and Experimental Design

A field experiment was conducted on abandoned vacant land in the Júndiz Industrial Park, located in the peri-urban belt of Vitoria-Gasteiz (42°50’N; 2°40’W, northern Spain, elevation 508 m a.s.l.). This area has a temperate Mediterranean climate with dry summers, cold winters, and a mean annual rainfall of 700–800 mm. Prior to the experiment, the soil surface was cleared by removing construction and demolition waste and other inert residues that were illegally dumped. Then, the soil was thoroughly mixed and leveled with a backhoe loader. Physicochemical analyses showed that the soil was slightly alkaline (pH > 8.5) and loamy, with low contents of topsoil organic matter (1.0%) and major nutrients (0.1% N DW^–1^ soil, 7.2 mg available P kg^–1^ DW soil, 85 mg available K^+^ kg^–1^ DW soil). Because of these low organic matter (OM) and nutrient contents, the soil was amended with compost (75 t ha^–1^) in order to facilitate plant growth and stimulate soil microbial activity. The compost was acquired from BIOCOMPOST de Álava U.T.E., a municipal solid-waste treatment plant located near the study area. The compost application rate was established following the recommendations regarding water content, OM content, C:N ratio, and metal contents of the Spanish legislation for organic fertilizers (Royal Decree 999/2017 on inorganic and organic fertilizers, transposition of EU Regulation No. 2003/2003) (Government of Spain, 2017). We used the recommended rate of application of a previous study performed in this area (Míguez et al., 2020). In this way, we ensure not only immediate correction of soil organic matter content (it was raised from 1 to 8.6%), but also its long-term positive effect without the need to intervene again and destroy the herbaceous community that could be potentially stablished. The compost was carefully mixed into the topsoil (0–30 cm depth), using a rotary tiller. The physicochemical characteristics of the compost-amended soil are detailed in Table 1. The compost amendment did not alter the soil pH but markedly increased N, P and K content, as expected.

**TABLE 1:** Soil physicochemical properties.

Total clay (%)	36.6
Coarse sand (%)	6.8
Fine sand (%)	13.8
Total silt (%)	42.8
Texture class (USDA)	Clay loam
pH (1:2.5)	8.5
Organic matter (% DW)	8.6
Total N (% DW)	0.4
Organic C / Organic N	13.3
Available P (mg kg^–1^ DW)	223.5
Total K^+^ (mg kg^–1^ DW)	3.6
Cadmium (mg kg^–1^ DW)	2.9
Copper (mg kg^–1^ DW)	44.8
Nickel (mg kg^–1^ DW)	19.5
Lead (mg kg^–1^ DW)	76.2
Chromium (mg kg^–1^ DW)	21.1
Zinc (mg kg^–1^ DW)	168.9
Cobalt (mg kg^–1^ DW)	14.1
Manganese (mg kg^–1^ DW)	176.6

In March 2017, one month after application of the compost, the following 6 treatments were performed in triplicate (18 plots of 5 m × 5 m): (1) no planting (control plot), where we followed the development of spontaneous vegetation (v); (2) alfalfa (*Medicago sativa*) (a); (3) non-inoculated hybrid black poplar (*Populus* × *canadensis*, hereafter ‘‘poplar’’) (Pv); (4) poplar inoculated with a commercial mycorrhizal inoculum (Piv); (5) non-inoculated poplar intercropped with alfalfa (Pa); and (6) inoculated poplar intercropped with alfalfa (Pia). Alfalfa (ecotype ‘‘Tierra de Campos’’) was sown in the corresponding plots at a seed rate of 20 kg ha^––1^. Unrooted poplar cuttings, provided by Biopoplar s.r.l.,^1^ were rooted in a greenhouse prior to transplantation in the field. The rooted cuttings were planted at a spacing of 1.5 m × 1.0 m. The commercial mycorrhizal inoculum (Micoplus, bioFlower Products) consisted of a consortium of ectomycorrhizae (*Rhizopogon* spp., *Scleroderma* spp., and *Pisolithus tinctorius* at 2,106 spores g^–1^) and endomycorrhizae (*Glomus intraradices* and *Glomus mosseae* at > 60 infective propagules g^–1^) attached to an inert medium bed. Following the manufacturer’s instructions, the mycorrhizae were inoculated in the corresponding treatments as follows: (i) during the rooting period of poplar cuttings in the greenhouse; (ii) in the field, just after the rooted poplar cuttings were planted; and (iii) three and (iv) 12 months after the rooted poplar cuttings were planted. The use of standardized mycorrhizal inoculum allows a more reproducible inoculation over both spatial and temporal scales. The experiment was monitored in the first (July 2017) and second (July 2018) growing seasons. A drought event occurred in August 2017.

### Determination of Soil Physicochemical Properties

In the first (July 2017) and second (2018) growing seasons, soil samples were collected from the top 20 cm, air-dried at room temperature, and passed through a 2-mm mesh. Physicochemical analyses of the soil samples were performed at the University of Santiago de Compostela (Spain), according to standard methods. The total metal concentrations in the soil samples were determined according to the microwave-heating EPA method 3050B for Inductively Coupled Plasma-Optical Emission Spectrophotometer (ICP-OES) analysis (U.S. Environmental Protection Agency (US-EPA), 1996). Soil samples for the determination of organic contaminants were taken just after implementation of the treatments (March 2017; t_0_) and at the end of the experiment (July 2018; t_f_). The concentrations of organic contaminants (i.e., total petroleum hydrocarbons, TPHs C10–C40; total polychlorinated biphenyls, PCBs; and polycyclic aromatic hydrocarbons, PAHs) were determined by a certified laboratory (SYNLAB Analytics & Services B.V., Rotterdam, Netherlands). Total C_10_-C_40_ hydrocarbon fractions were analyzed according to ISO 16703 (2004) standard using hexane/acetone extraction and Gas Chromatography-Flame Ionization Detector. The PCBs and PAHs contents were determined in accordance with an internal method accredited by the Dutch RvA (Raad voor Accreditatie) and ISO 18287 (2006) standard, respectively, followed by Gas Chromatography-Mass Spectrometry (GC-MS) detection.

### Determination of Plant Parameters

Plant diversity, biomass, and ecophysiological parameters were determined 3 months after the treatment implementation (first growing season, July 2017) and at the end of the experiment (second growing season, July 2018).

For the study of plant diversity and biomass, three quadrats, each measuring 0.5 m × 0.5 m (0.25 m^2^) were placed at random in each plot. All vascular plant species within the quadrats were identified to species level. Then, shoots were removed and weighed (FW). Subsequently, the plants were dried at 40°C for 48 h and the dry mass (DW) was calculated.

Three fully expanded leaves from three poplar individuals in each replicate plot were sampled. The following biometric measurements were taken for these trees: (i) leaf dry weight (DW); (ii) specific leaf area (SLA), calculated as the coefficient between leaf area and DW; and (iii) total branch length, as the sum of the lengths of all branches. Leaves were maintained in the laboratory in 100% relative humidity, room temperature, and darkness for 12 h to mimic pre-dawn conditions. The maximum photochemical efficiency of PSII (F_v_/F_m_) was estimated using a portable fluorimeter (FluorPen FP100, Photon Systems Instruments). The maximum chlorophyll (Chl) fluorescence yield (F_m_) was induced with a saturating pulse. The minimum (Chl) fluorescence (F_0_) was recorded at low measuring light intensities. F_v_/F_m_ was calculated as (F_m_ – F_0_)/F_m_ (García-Plazaola et al., 1999). The contents of chlorophylls, carotenoids, xanthophylls [violaxanthin (V), antheraxanthin (A), and zeaxanthin (Z)], and tocopherols were determined using the ultra-rapid UHPLC method, following Lacalle et al. (2020). Briefly, six discs (7.35 mm diameter) were collected from the youngest fully expanded leaf, frozen in liquid nitrogen, and stored at –80°C until analysis. The leaf discs were homogenized using a Tissue-Tearor in 1 mL pure acetone, the mixture was centrifuged at 13,200 × *g* for 20 min, and 1.5 mL of the supernatant was filtered through a PTFE filter (0.2 μm) and analyzed using the Acquity^TM^ UHPLC H-Class system (Waters^®^, Milford, MA, United States).

### Determination of Soil Microbial Properties

In July 2017 and July 2018, soil samples were collected to determine the soil basal respiration (BR, indicator of soil microbial activity), substrate-induced respiration (SIR, indicator of potentially active microbial biomass), and bacterial functional diversity, as described by Epelde et al. (2008). Bacterial functional diversity was determined using Biolog EcoPlates^TM^ (Biolog Inc., Hayward, CA, United States), following Epelde et al. (2008). Average well color development (AWCD), Shannon’s diversity index (H′), and substrate-consumption activity (SCA = area under the curve) at the mid-exponential growth phase (after 40 h of incubation) were calculated from the Biolog data.

At the end of the experiment (July 2018), soil samples were sieved to < 2 mm prior to the determination of total bacteria and total fungi (according to gene copy abundance values), soil enzyme activities, and microbial structural diversity, through amplicon sequencing.

For the quantification of total bacteria and fungi, DNA was extracted in triplicate from 0.25 g DW soil, using the Power Soil DNA Isolation Kit (MO Bio Laboratories, Carlsbad, CA, United States). Prior to DNA extraction, the soil samples were washed twice in 120 mM K_2_HPO_4_ (pH 8.0) to remove extracellular DNA (Kowalchuk et al., 1997). The amount of DNA in the samples was determined in a ND-1000 spectrophotometer (Thermo-Scientific, Wilmington, DE, United States). Real-time qPCR was carried out to determine total bacterial (16S rRNA) and fungal (internal transcribed spacer, ITS) gene-copy abundances for the estimation of total bacteria and fungi, respectively, according to Epelde et al. (2014).

The activities of the enzymes β-glucosidase (EC 3.2.1.21), β-glucosaminidase (EC 3.2.1.30), arylsulfatase (EC 3.1.6.8), acid phosphatase (EC 3.1.3.2), L-Ala-aminopeptidase (EC 3.4.11.12), and L-Leu-aminopeptidase (EC 3.4.11.1) were measured using fluorogenic substrates in microwell plates, following ISO/TS 22939 (International Organization for Standardization (ISO), 2010).

In order to assess the effect of treatments on the soil microbial structural diversity and composition, amplicon libraries were prepared using a dual indexing tag-tailed approach (D’Amore et al., 2016). The following sequence-specific primer pairs were used (Lanzén et al., 2016): 519F (CAGCMGCCGCGGTAA) from Øvreås et al. (1997) and 806R (GGACTACHVGGGTWTCTAAT) from Caporaso et al. (2012) for targeting the prokaryotic 16S rRNA hypervariable region V4; and ITS1F (CTTGGTCATTTAGAGGAAGTAA) and ITS2R (GCTGCGTTCTTCATCGATGC) (McGuire et al., 2013) for targeting the fungal ITS1 region. Libraries were sequenced by the Tecnalia Corporation (Miñano, Spain), using an Illumina MiSeq platform with the V2 kit and pair-ended 2 × 250 nt. The generated paired-end reads were merged, quality-filtered (i.e., primer trimming, removal of singletons and chimeric sequences), and grouped into operational taxonomic units (OTUs), following Lanzén et al. (2016). Finally, taxonomic assignments were made using CREST and SilvaMod v128 (Lanzén et al., 2012).

### Statistical Analyses

All data are expressed as mean ± standard error (*n* = 3). First, data distribution and homoscedasticity were checked using the Kolmogorov-Smirnov and Levene tests, respectively. All original data showed normal distributions. Statistically significant effects (*p* < 0.05) of the treatments on plant and soil microbial (BR, SIR, bacterial functional diversity) parameters were determined using one-way analysis of variance (ANOVA). A three-way ANOVA was applied to examine the potential interactive effects of (1) time (2-year temporal evolution), (2) mycorrhizal inoculation, and (3) intercropping alfalfa-poplar on the above-mentioned parameters at a 0.05 level of significance. The statistical analysis was carried out using IBM SPSS Statistics for Windows (Version 24; IBM Corp., Armonk, NY, United States). Principal components analysis (PCA), executed with Canoco 5 software (ter Braak and Smilauer, 2012), was used to identify possible relationships among plant and soil microbial parameters.

The effects of the treatments on soil enzyme activities and total bacteria and fungi were analyzed by means of one-way ANOVA and Duncan’s multiple range test, using the agricolae package in the R software (Version 3.3.2). The values of all soil enzyme activities were used for the calculation of overall enzyme activity (OEA), following Epelde et al. (2014):

$$\mathrm{OEA}=10^{\log\,m}+\frac{\sum_{i=1}^{n}\left({\mathrm{logn}}_{i}-\mathrm{logm}\right)}{n}$$

where *m* is the reference value (set to 100% for the mean value of each enzyme activity in the control soil) and *n* corresponds to the measured values for each enzyme activity as a percentage of the reference value. Soils from the plots where spontaneous vegetation was growing (v) were considered as controls. Significant differences in OEA values were analyzed by means of one-way ANOVA, with Duncan’s post-hoc comparisons. In all tests, a *p*-value <0.05 was considered to be statistically significant.

Regarding the microbial structural diversity data, calculation of α-diversity indices (R richness: rarefied richness, H′: Shannon’s index, J′: Pielou’s evenness), multivariate statistics, and visualization of sequencing data from the 16S rRNA and ITS amplicon libraries were performed with the vegan package in the R software (Oksanen et al., 2015). Rarefied richness estimates, interpolating the expected richness at the lowest sample-specific sequencing depth, were used to compensate for variations in read numbers across samples (Lanzén et al., 2016). The function decostand was used to transform OTU distributions into relative abundances. Bray-Curtis dissimilarity matrices were calculated to compare the prokaryotic and fungal community composition between samples. These matrices were further used to perform non-metric multidimensional scaling (NMDS) with the function metaMDS. Permutational analyses of variance (PERMANOVA) were performed to assess the impacts of the treatments on the soil prokaryotic and fungal community composition, using the function adonis. Pairwise analyses of the relative abundances of each taxon at order level, representing more than 0.1% of the total reads for both prokaryotic and fungal communities, were performed by means of two-way ANOVA in order to detect significant differences among experimental factors.

## Results

### Contaminant Degradation

The soil from the abandoned vacant land contained low concentrations of heavy metals (Cd, Cu, Zn, Pb) that did not exceed the Critical Reference Values for Ecosystem Protection established by Law 4/2015 of the Basque Country for agricultural settings (Government of the Basque Country, 2015). Nonetheless, although total metal concentrations did not exceed regulatory limits, we decided to quantify the effects of the treatments on these concentrations. At the end of the experiment, the concentrations of Cd, Cu, and Zn had decreased in all the treatments (75–88% reduction of Cd, 33–73% reduction of Cu, and 60–82% reduction of Zn), while the concentration of Pb was virtually unchanged (probably due to the very low mobility and availability of this metal in soil). The highest decrease in the total concentrations of the most mobile metals (Cd and Zn) in soil could be due to leaching and/or plant uptake. In any case, total metal concentrations were very low for a contaminated soil. As indicated above, the soil was contaminated with TPHs, PCBs, and PAHs (Table 2). As is often the case in similar contaminated sites, this peri-urban vacant land had a highly heterogeneous distribution of soil contaminants among the plots. Therefore, in order to assess the effects of the treatments on contaminant degradation, we compared those plots with similar concentrations of contaminants just after treatment implementation (March 2017; t_0_) *vs.* at the end of the experiment (July 2018; t_f_).

**TABLE 2:** Concentration of soil organic contaminants.

**Contaminant (mg kg^–1^)**	**Treatment**						
	**Sampling time**	**a**	**Pa**	**Pia**	**Pv**	**Piv**	**v**
TPH (C10–C40)	t_0_	220.0	130.0	85.0	70.0	130.0	90.0
	t_f_	108.3	173.3	83.3	146.7	118.3	105.0
Total PCBs	t_0_	17.0	8.4	9.0	22.0	15.0	9.0
	t_f_	16.7	8.9	18.7	15.0	13.7	7.4
Benzo[a]pyrene	t_0_	0.06	0.03	0.05	0.09	0.04	0.02
	t_f_	0.04	0.04	0.06	0.05	0.04	0.04
Benzo[b]fluoranthene	t_0_	0.09	0.05	0.08	0.14	0.06	0.03
	t_f_	0.07	0.06	0.1	0.08	0.07	0.07

*Concentrations (mg kg^–1^) of Total Petroleum Hydrocarbons (TPH), Total PCBs, and PAHs (benzo[a]pyrene and benzo[b]fluoranthene) before (t_0_) and after (t_f_) application of the following treatments: a, planting of alfalfa; Pa, intercropping of non-mycorrhizal poplars with alfalfa; Pia, intercropping of mycorrhizal poplars with alfalfa; Pv, planting of non-mycorrhizal poplars with spontaneous vegetation; Piv, planting of mycorrhizal poplars with spontaneous vegetation; v, spontaneous vegetation (unplanted control). For reasons of spatial heterogeneity of organic contaminants, only those plots under different treatments with similar concentrations were compared. Critical Reference Values for Ecosystem Protection according to Basque legislation (mg kg^–1^): TPH = 50; PCB = 0.01; Benzo[a]pyrene = 0.02; and Benzo[b]fluoranthene = 0.2.*

At t_0_, TPHs were the most abundant contaminants in the soil, with concentrations ranging from 70–200 mg kg^–1^, compared to PCBs (0–22 mg kg^–1^) and PAHs (benzo[a]pyrene: 0.02–0.09 mg kg^–1^; benzo[b]fluoranthene: 0.03–0.14 mg kg^–1^) (Table 2). Overall, all these organic contaminants exceeded their respective Critical Reference Values established by the above-mentioned Basque legislation, and accordingly they must be remediated.

We did not observe phytotoxic symptoms caused by organic contaminants on plant communities developed in our studied plots, in comparison with the vegetation of non-contaminated sites within the study area. This could arise due to the low bioavailability of organic contaminants in soils resulted from decade-long aging processes. After the end of the experiment (t_f_), alfalfa (a) proved to be the most effective treatment in terms of TPH and PAH degradation (Table 2). Mycorrhizal poplars, when planted alone, stimulated the degradation of TPHs, while non-mycorrhizal poplars enhanced PCB and PAH degradation. The low contaminant concentrations and, above all, their highly heterogeneous distribution in the soil hampered interpretation of the TPH degradation values. Likewise, the extremely low PAH concentrations (benzo[a]pyrene and benzo[b]fluoranthene) in some treatments, very close to the detection limit of the analytical technique used here for their quantification (0.02 mg kg^–1^) and also close to the corresponding Critical Reference Values (0.02 and 0.2 mg kg^–1^ for benzo[a]pyrene and benzo[b]fluoranthene, respectively) further complicated the interpretation of the data.

### Plant Performance and Diversity

**FIGURE 1:**
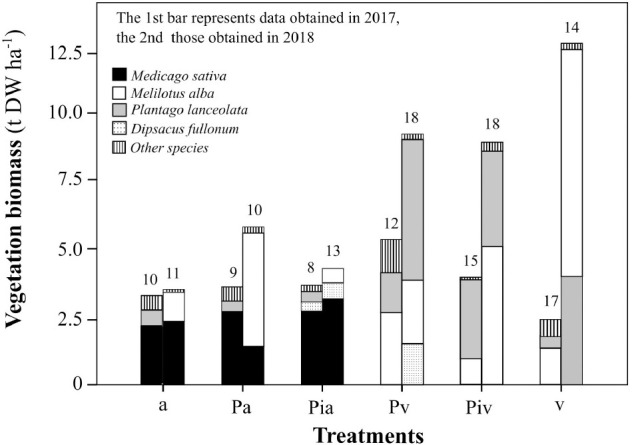
Biomass of herbaceous vegetation. Treatments: a, planting of alfalfa; Pa, intercropping of non-mycorrhizal poplars with alfalfa; Pia, intercropping of mycorrhizal poplars with alfalfa; Pv, planting of non-mycorrhizal poplars with spontaneous vegetation; Piv, planting of mycorrhizal poplars with spontaneous vegetation; and v, spontaneous vegetation (unplanted control). Each stacked bar represents the total biomass of all the species found in each treatment. Different colors represent the biomass of the most abundant plant species. Numbers above bars indicate the total number of species found in each treatment.

Data on the biomass of the most abundant plant species, as well as the values for plant richness, are shown in Figure 1. All plots (planted and unplanted with alfalfa and/or poplar) were colonized by spontaneous vegetation. Some spontaneous plant species (*Melilotus albus, Plantago lanceolata*, and *Dipsacus fullonum)* were present in all plots and contributed substantially to the total biomass.

The presence of alfalfa, whether in a monoculture or in an intercropping system, limited the plant biodiversity. Plots where alfalfa had been sown showed the lowest levels of plant diversity, and this remained the case until the experiment ended. The highest levels of plant diversity were observed in plots where poplars (whether or not inoculated with the commercial mycorrhizal inoculum) had been transplanted, but only in the absence of alfalfa. This plant biodiversity was highest at the end of the experiment. Unplanted control plots were colonized by spontaneous vegetation and showed high plant diversity, dominated mainly by *M. albus*. This leguminous species colonized all plots, reaching its highest biomass in the unplanted control treatment. The significant growth of *M. albus* can probably explain the decrease in alfalfa biomass observed in the poplar/alfalfa intercropping system (Pa treatment) at the end of the experiment, most likely as a consequence of competition between these two leguminous species.

**FIGURE 2:**
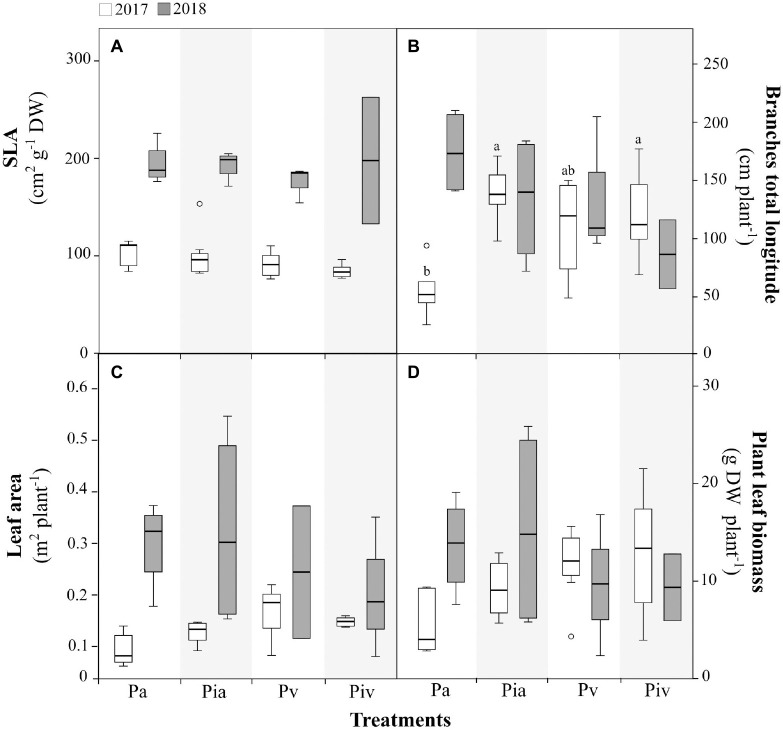
Biometric parameters in poplar (Populus × canadensis) saplings in 2017 (open bars) and 2018 (closed bars). Treatments: Pa, intercropping of non-mycorrhizal poplars with alfalfa; Pia, intercropping of mycorrhizal poplars with alfalfa; Pv, planting of non-mycorrhizal poplars with spontaneous vegetation; and Piv, planting of mycorrhizal poplars with spontaneous vegetation. **(A)** Specific Leaf Area (SLA); **(B)** Total branch length; **(C)** Plant leaf area; **(D)** Plant leaf biomass. Small letters indicate significant differences between treatments in 2017 (*p* < 0.05). In 2018, there were no statistically significant differences between treatments. Each bar represents the mean ± SE (*n* = 3).

**FIGURE 3:**
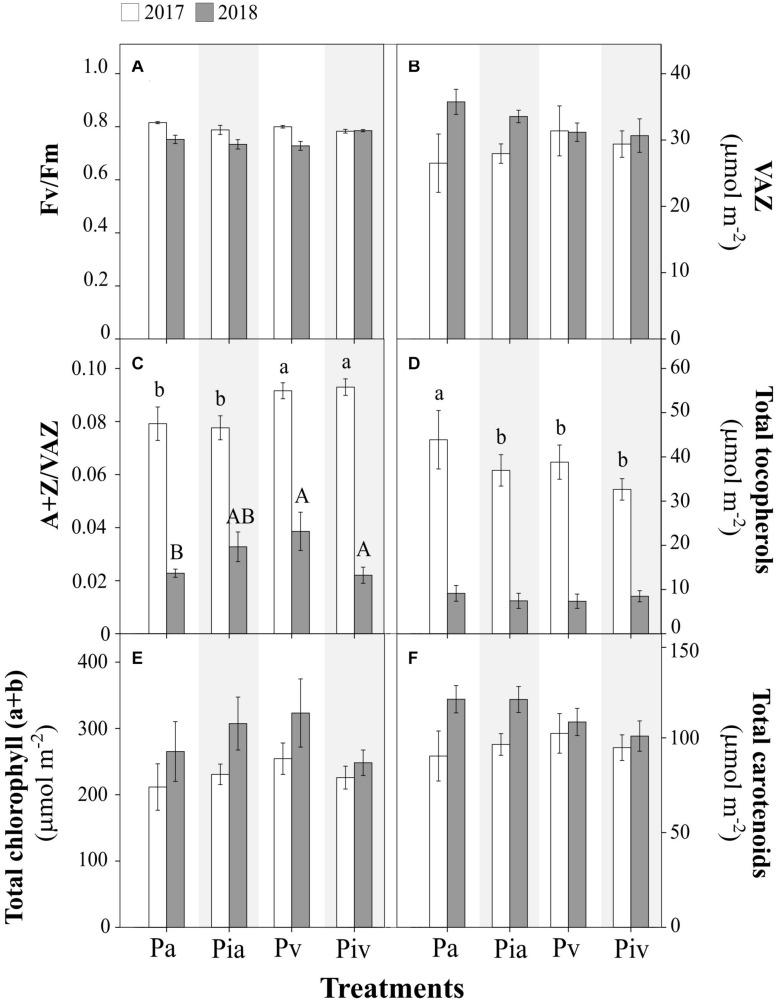
Physiological parameters in poplar (Populus × canadensis) sapling leaves in 2017 (open bars) and 2018 (closed bars). Treatments: Pa, intercropping of non-mycorrhizal poplars with alfalfa; Pia, intercropping of mycorrhizal poplars with alfalfa; Pv, planting of non-mycorrhizal poplars with spontaneous vegetation; Piv, planting of mycorrhizal poplars with spontaneous vegetation. **(A)** Photochemical efficiency of PSII (F_v_/F_m_); **(B)** Total xanthophyll cycle pigments (VAZ); **(C)** De-epoxidation state of xanthophyll cycle pigments (A+Z/VAZ); **(D)** Total tocopherols; **(E)** Total chlorophyll; **(F)** Total carotenoids. Small and capital letters indicate significant differences between treatments in 2017 and 2018, respectively (*p* < 0.05). Each bar represents the mean ± SE (*n* = 3).

The performance of poplar trees during the first (July 2017) and second (July 2018) growing seasons is shown in Figures 2, 3. Regarding the biometric parameters measured here (Figure 2), the most important finding was probably the interspecific competition observed in the poplar/alfalfa intercropping treatment (Pa) in July 2017. More specifically, sowing alfalfa reduced the biomass of poplar branches in the first growing season (Figure 2B), but this adverse effect was not observed in the Pia treatment, where poplars were inoculated with the mycorrhizal inoculum and intercropped with alfalfa. No other differences in biometric parameters were observed in the first growing season. However, the levels of specific leaf area (Figure 2A) and total photosynthetic area (Figure 2C) in poplar trees increased in all treatments during the second growing season, indicating that the post-planting period is of critical importance for poplar tree performance.

Regarding ecophysiological plant parameters, such as photochemical efficiency F_v_/F_m_ (Figure 3A), VAZ content (Figure 3B), A+Z/VAZ de-epoxidation index (Figure 3C), tocopherol content (Figure 3D), total chlorophyll (Figure 3E), and carotenoids (Figure 3F), no significant differences were detected among the treatments. As was observed for some biometric parameters (Figures 2A,C), large differences in certain photoprotection indicators were observed between July 2017 and July 2018, i.e., the de-epoxidation index (Figure 3C) and the content of tocopherols (Figure 3D) were much higher in July 2017 than in July 2018. This observation reinforces the need to pay special attention to the initial steps in transplanting cuttings, in order to avoid possible episodes of plant stress, especially if poplar trees are to be intercropped with alfalfa.

### Soil Microbial Properties

**FIGURE 4:**
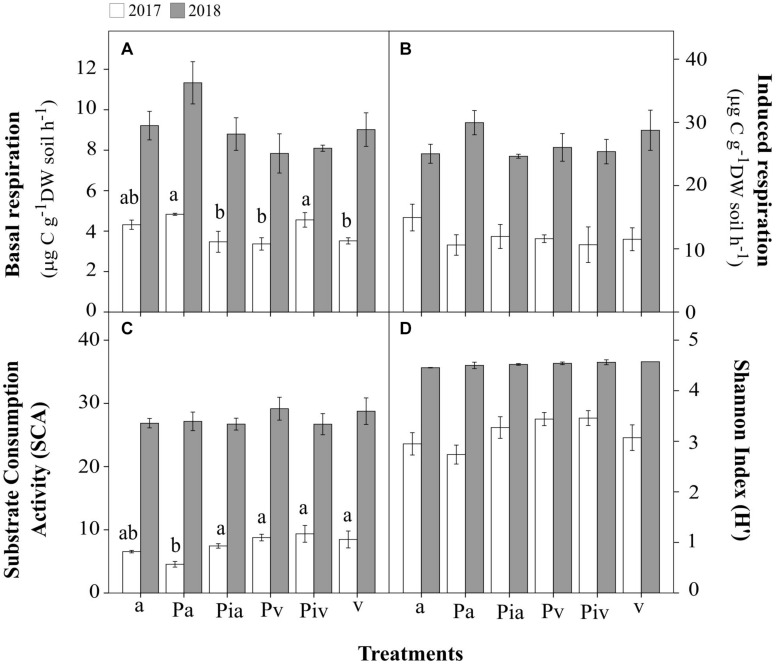
Soil microbial activity, biomass, and bacterial functional diversity in 2017 (open bars) and 2018 (closed bars). Treatments: a, planting of alfalfa; Pa, intercropping of non-mycorrhizal poplars with alfalfa; Pia, intercropping of mycorrhizal poplars with alfalfa; Pv, planting of non-mycorrhizal poplars with spontaneous vegetation; Piv, planting of mycorrhizal poplars with spontaneous vegetation; v, spontaneous vegetation (unplanted control). Small letters indicate significant differences among treatments in 2017 (p < 0.05). **(A)** microbial activity in terms of basal respiration; **(B)** potentially active microbial biomass in terms of substrate-induced respiration; **(C)** Substrate Consumption Activity (SLA); **(D)** Shannon Index (H′) for bacterial functional diversity. Small letters indicate significant differences between treatments in 2017 (*p* < 0.05). In 2018, there were no statistically significant differences between treatments. Each bar represents the mean ± SE (*n* = 3).

**FIGURE 5:**
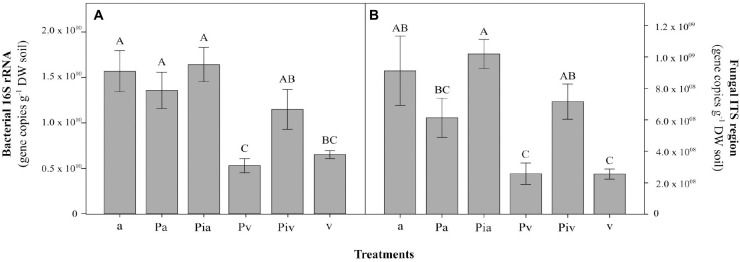
Bacterial **(A)** and fungal **(B)** gene copy abundances at the end of the experiment (2018, t_f_). Treatments: a, planting of alfalfa; Pa, intercropping of non-mycorrhizal poplars with alfalfa; Pia, intercropping of mycorrhizal poplars with alfalfa; Pv, planting of non-mycorrhizal poplars with spontaneous vegetation; Piv, planting of mycorrhizal poplars with spontaneous vegetation; v, spontaneous vegetation (unplanted control). Capital letters indicate significant differences between treatments according to one-way ANOVA and Duncan’s MRT (*p* < 0.05). Each bar represents the mean ± SE (*n* = 3).

In the first growing season (July 2017), the levels of basal respiration (Figure 4A), substrate-induced respiration (Figure 4B), and bacterial functional diversity (Figures 4C,D) were lower than in July 2018. Soils in the poplar/alfalfa intercropping system (Pa treatment) showed significantly lower substrate-consumption activity (SCA) compared to the plots with poplar trees and spontaneous vegetation (Pv and Piv treatments). In the second growing season (July 2018), however, the levels of all these parameters increased 2–3-fold over the levels in July 2017. In July 2018, soils where alfalfa had been sown showed significantly higher abundances of bacterial (96%, Figure 5A; *p* < 0.001) and fungal (107%, Figure 5B; *p* < 0.01) gene copies compared to treatments with spontaneous vegetation, with the exception of the plots where mycorrhizal poplars were intercropped with alfalfa (Pia treatment; Figure 5B; *p* < 0.05).

**FIGURE 6:**
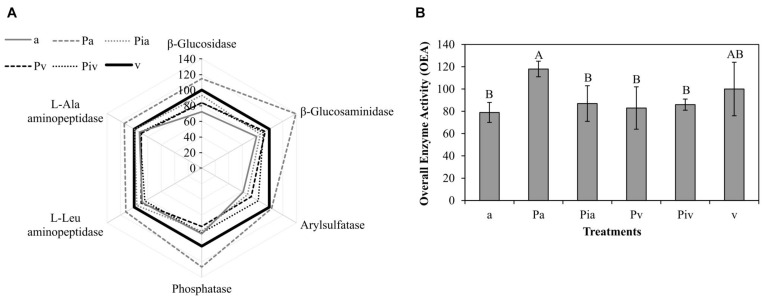
Soil enzyme activities at the end of the experiment (2018, t_f_). Treatments: a, planting of alfalfa; Pa, intercropping of non-mycorrhizal poplars with alfalfa; Pia, intercropping of mycorrhizal poplars with alfalfa; Pv, planting of non-mycorrhizal poplars with spontaneous vegetation; Piv, planting of mycorrhizal poplars with spontaneous vegetation; v, spontaneous vegetation (unplanted control). **(A)** Sunray plot of enzyme activities, where 100% corresponds to the mean value obtained for each enzyme activity in the unplanted control soil (v treatment). **(B)** Overall Enzyme Activity (OEA), which integrates all measured enzyme activities into a single value. Capital letters indicate significant differences between treatments for the OEA value, according to one-way ANOVA and Duncan’s MRT (*p* < 0.05). Each bar represents the mean ± SE (*n* = 3).

Soil enzyme activities in general showed no significant differences among treatments (Figure 6); only the Pa treatment showed significantly higher activities. Indeed, the highest OEA levels (Figure 6) were detected in soils under the non-mycorrhizal poplars intercropped with alfalfa (Pa treatment). Intercropping significantly (*p* < 0.05) increased OEA values compared to monoculture plots, regardless of whether the poplar trees were inoculated with mycorrhizae (Figure 6).

Concerning soil microbial structural diversity, after singleton removal and quality filtering, amplicon sequencing resulted in 1,407,301 prokaryotic (16S rRNA) reads, which were clustered into 7,544 OTUs; while ITS amplicon sequencing resulted in 1,726,907 fungal (ITS1) reads, clustered into 1,233 OTUs, at a 3% dissimilarity level. The number of reads correlated significantly with total OTU richness for the 16S rRNA data (*p* < 0.001), suggesting an insufficient sequencing effort to obtain full coverage of the soil prokaryotic diversity. No significant correlation between the number of ITS1 reads and OTU richness was observed. However, considerable differences in the number of ITS1 reads were observed between samples: 72,753 and 121,363 ITS1 reads in the samples with the lowest and highest number of reads, respectively. At the end of the experiment (July 2018), no significant differences were observed between treatments regarding the values of the α-diversity indices for soil prokaryotic and fungal communities (Table 3). Plots where alfalfa had been sown showed significantly lower levels of prokaryotic (*p* < 0.05) and fungal (*p* < 0.001) rarefied richness, as well as significantly lower levels of fungal H′ and J′ (*p* < 0.05 for both), in comparison with the plots where spontaneous plant species established themselves.

**TABLE 3:** Structural diversity of soil microbial communities.

**Treatment**	**Prokaryotic (16S rRNA)**			**Fungal (ITS)**		
	**R richness**	**H′**	**J′**	**R richness**	**H′**	**J′**
a	3147 ± 147^AB^	6.3 ± .3 ^A^	0.77 ± .77 ^A^	400 ± 00 ^E^	3.23 ± .23 ^B^	0.53 ± .53 ^A^
Pa	3035 ± 035 ^B^	6.2 ± .2 ^A^	0.77 ± .77 ^A^	453 ± 53 ^CD^	3.43 ± .43 ^AB^	0.55 ± .55 ^A^
Pia	3155 ± 155^AB^	6.3 ± .3 ^A^	0.78 ± .78 ^A^	426 ± 26 ^DE^	3.40 ± .40 ^AB^	0.55 ± .55 ^A^
Pv	3205 ± 205 ^AB^	6.2 ± .2 ^A^	0.75 ± .75 ^A^	521 ± 21 ^AB^	3.70 ± .70 ^A^	0.59 ± .59 ^A^
Piv	3223 ± 223^AB^	6.4 ± .4 ^A^	0.78 ± .78 ^A^	480 ± 80 ^BC^	3.46 ± .46 ^AB^	0.56 ± .56 ^A^
v	3280 ± 280 ^A^	6.1 ± .1 ^A^	0.75 ± .75 ^A^	537 ± 37 ^A^	3.70 ± .70 ^A^	0.59 ± .59 ^A^

*Rarefied richness (R richness), Shannon’s index (H′), and Pielou’s evenness (J′) at the end of the experiment (t_f_, 2018). Treatments: a, planting of alfalfa; Pa, intercropping of non-mycorrhizal poplars with alfalfa; Pia, intercropping of mycorrhizal poplars with alfalfa; Pv, planting of non-mycorrhizal poplars with spontaneous vegetation; Piv, planting of mycorrhizal poplars with spontaneous vegetation; v, spontaneous vegetation (unplanted control). Different capital letters indicate significant differences between treatments (*p* < 0.05).*

Regarding the soil microbial community composition (OTU profiles), NMDS analyses based on the Bray-Curtis dissimilarity matrix showed that both prokaryotic and fungal communities from the same soil samples tended to cluster together, indicating similar distribution patterns. On the other hand, at the end of the experiment (July 2018), the different treatments were separated according to both 16S rRNA and ITS amplicon data (PERMANOVA *p* < 0.001) (Supplementary Figure 1), indicating that they all had a strong effect on the composition of the soil prokaryotic and fungal communities. Unplanted control plots with their spontaneous vegetation showed the most differentiated soil prokaryotic and fungal communities, in both cases constituting individual clusters markedly separated from the rest.

In relation to soil microbial taxonomic classification, more than 88% of the 16S rRNA and ITS sequences were classified to order rank (for family rank, the classification of the ITS sequences dropped to 73%). Since the use of a deeper taxonomic resolution may not compensate for the loss of information regarding the rarest OTUs, we chose to use the order rank for our taxonomic analysis. At the order rank, the treatments were similar regarding the identity of the 30 most dominant prokaryotic (Supplementary Figure 2) and fungal (Supplementary Figure 3) taxa. Nevertheless, the abundances of these dominant taxa differed somewhat among treatments. At the end of the experiment, the most abundant bacterial orders were Xanthomonadales and Flavobacteriales (9.4 and 9.3% of the total prokaryotic community, respectively) in all the soils (Supplementary Figure 2). Nonetheless, the relative abundance of these two orders was significantly lower in the soils where spontaneous vegetation was established (7.0 and 4.0%, respectively). In contrast, the soils with spontaneous vegetation showed higher abundances of Sphingomonadales (8.0%) and Pseudomonadales (8.3%) compared to the planted soils (6.3 and 2%, respectively) (Supplementary Figure 2). Regarding fungal communities, plots with spontaneous vegetation showed lower abundances of Ascomycota SH195297.07FU and Pleosporales (5.1 and 3.1% of the fungal community, respectively), compared to planted plots (12.8 and 8.3% of the fungal community, respectively), while showing a significantly higher abundance of Agaricales (13.5% *vs* 1.8%) (Supplementary Figure 3).

**FIGURE 7:**
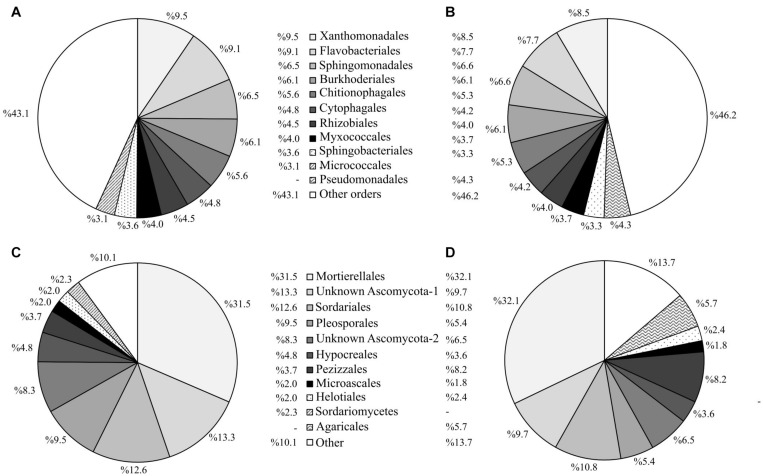
Abundance profiles of the 10 most abundant prokaryotic and fungal taxa at order level for plots under the experimental factor “alfalfa sowing” at the end of the experiment. Abundance of prokaryotic taxa in plots with **(A)** and without **(B)** alfalfa. Abundance of fungal taxa in plots with **(C)** and without **(D)** alfalfa.

**FIGURE 8:**
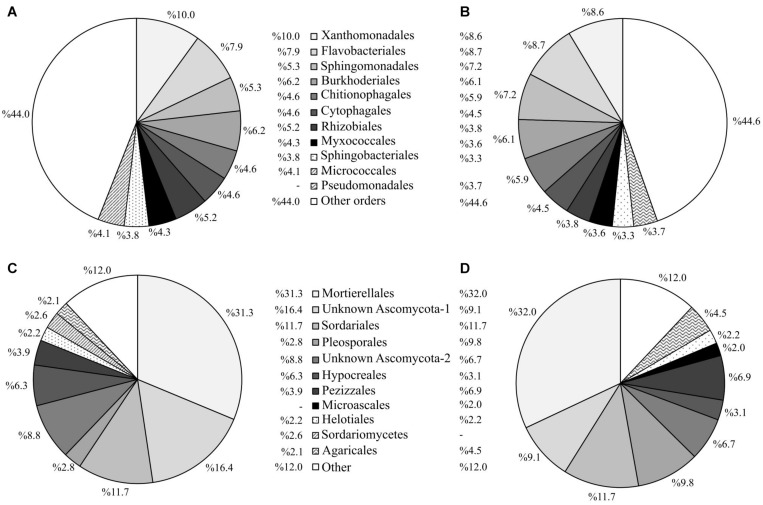
Abundance profiles of the 10 most abundant prokaryotic and fungal taxa at order level for plots under the experimental factor “mycorrhizal inoculation” at the end of the experiment. Abundance of prokaryotic taxa in plots with mycorrhizal **(A)** and non-mycorrhizal **(B)** poplars. Abundance of fungal taxa in plots with mycorrhizal **(C)** and non-mycorrhizal **(D)** poplars.

The 10 most abundant prokaryotic and fungal taxa in plots where alfalfa had been sown comprised 57 and 90% of the total community, respectively, compared to 54 and 86% for the unplanted control plots (Figures 7, 8). This result is in accordance with the lower values of microbial α-diversity in soils where alfalfa had been sown (Table 3). The order Pseudomonadales was not among the 10 most abundant prokaryotic taxa in the alfalfa (a) treatment (comprising only 1.8% of the total prokaryotic community), while in the unplanted control, this order comprised 4.3% of the total prokaryotic community (Figure 7A). Regarding soil fungal communities, the presence of alfalfa increased the abundances of the unidentified order Ascomycota SH195297.07FU and the order Pleosporales, compared to the unplanted control, which in turn showed higher abundances of the orders Pezizales and Agaricales (Figure 7B).

Mycorrhizal inoculation of poplar trees significantly increased the abundance of the prokaryotic orders Xanthomonadales, Rhizobiales, and Micrococcales in comparison to soils where non-mycorrhizal poplars were planted (Figure 8A). On the other hand, mycorrhizal inoculation significantly reduced the abundances of the orders Sphingomonadales and Chitinophagales. Regarding the most abundant fungal taxa, mycorrhizal inoculation increased the abundances of two unidentified Ascomycota orders (SH195297.07FU and SH210351.07FU), which together comprised 25.2% of the total fungal community (Figure 8B). Within the non-mycorrhizal poplar plots, these two unidentified Ascomycota orders were less abundant, comprising 15.8% of the total fungal community. Mycorrhizal inoculation also led to a reduction in abundance of the orders Pleosporales, Pezizales, and Agaricales (Figure 8B). Mycorrhizal inoculation resulted in a higher number of significant differences in the abundance of prokaryotic taxa, with 15 taxa showing significant differences between inoculated and non-inoculated poplar plots (Supplementary Table 1). In comparison with prokaryotic taxa, the abundance of fungal taxa showed fewer differences among treatments, irrespective of the presence of alfalfa or mycorrhizal inoculation (Supplementary Table 1).

**FIGURE 9:**
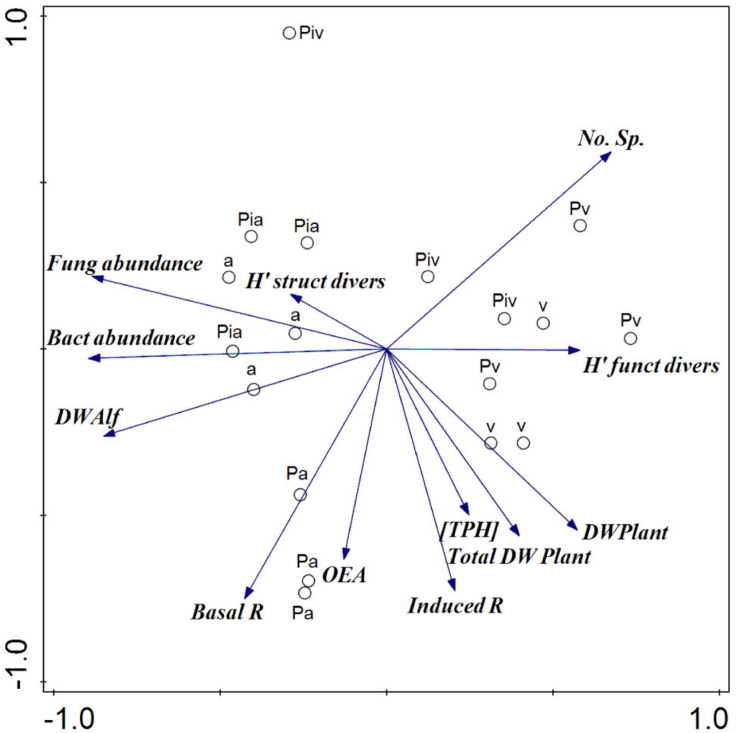
Principal components analysis of plant and soil microbial parameters. PC1 and PC2 accounted for 33.2 and 23.7% of the explained variance, respectively. [TPH], concentration of total petroleum hydrocarbons; No. Sp, number of species; DW Alf, dry weight of alfalfa; DW Plant, biomass in dry weight of spontaneous vegetation; Total DW Plant, biomass in dry weight of alfalfa and spontaneous vegetation; Induced R, substrate-induced respiration; Bact abundance, bacterial abundance by qPCR; Fung abundance, fungal abundance by qPCR; Basal R, basal respiration; OEA, Overall Enzyme Activity; H′ funct divers, Shannon’s bacterial functional diversity; H′ struct divers, Shannon’s microbial structural diversity. Treatments: a, planting of alfalfa; Pa, intercropping of non-mycorrhizal poplars with alfalfa; Pia, intercropping of mycorrhizal poplars with alfalfa; Pv, planting of non-mycorrhizal poplars with spontaneous vegetation; Piv, planting of mycorrhizal poplars with spontaneous vegetation; v, spontaneous vegetation (unplanted control).

The PCA (Figure 9) indicated a marked separation of treatments with respect to the values of plant and soil microbial parameters. The presence of alfalfa and mycorrhizal inoculation (main components) explained 60% of the total variance in these parameters. Spontaneous vegetation appeared to be associated with plant diversity and biomass, as well as with soil substrate-induced respiration and bacterial functional diversity, toward the positive region of PC1; while plots where alfalfa had been sown (in monoculture or intercropping) appeared more closely linked to microbial activity and abundance parameters, toward the negative region of PC1. In contrast, the mycorrhizal poplars grouped along the positive region of PC2 and appeared to be closely associated with the microbial structural diversity data.

## Discussion

### Contaminant Degradation

The presence of low concentrations of soil contaminants (although above regulatory limits, so that the soils are legally considered contaminated), together with low levels of contaminant availability in soil (due to the intrinsic persistence or recalcitrance of the contaminants, or to very long aging times) can greatly hinder the effectiveness of remediation strategies (Mukherjee et al., 2014). In this respect, when soil organic contaminants have low water solubility and bioavailability, their microbial degradation is severely limited. In our study, the very long aging times, together with the low concentrations and hydrophobicity of the organic contaminants (TPHs, PCBs, and PAHs) probably resulted in their being tightly bound and retained in the soil matrix, thus limiting microbial degradation (Table 2). The short time elapsed since treatment implementation and the drought event that occurred in August 2017 likely also contributed to the low effectiveness of the treatments in reducing contaminant concentrations. Longer-term studies are needed to puzzle out whether these organic contaminants will be ultimately degraded or not. A thorough examination of the effects of mycorrhizal inoculation and intercropping on the phytoremediation of soils contaminated with organic compounds, especially at low concentrations, requires much additional research. After all, the majority of investigations dealing with the effect of mycorrhizal inoculation on phytoremediation efficiency have been conducted in metal-contaminated soils. Inoculation of mycorrhizal fungi has been used to improve the phytoremediation of a variety of organic contaminants, such as TPHs (Bois et al., 2005; Gunderson et al., 2007), PAHs (Joner et al., 2006), and explosives (Thompson et al., 1998; Thompson and Polebitski, 2010). However, in most cases, in agreement with our results, a low phytoremediation efficiency was observed. According to Coninx et al. (2017), the effect of mycorrhizal inoculants is highly variable, as it depends on the nature of the contaminants, the specific mycorrhizal species, the plant species, the soil environmental conditions, and/or their potential interactions with the soil matrix and other soil organisms, etc. Furthermore, due to the well-known hydrophobicity and low bioavailability of TPHs (Garousin et al., 2021), PCBs (Ancona et al., 2017), and PAHs (Abraham et al., 2002), other strategies, such as the application of surfactants, could facilitate rhizodegradation processes, as these compounds can enclose colloidal particles of the organic contaminants, making them soluble in the aqueous phase and thereby increasing their bioavailability and hence their biodegradability (Martienssen and Schirmer, 2010).

### Plant Performance and Diversity

Plant growth and diversity are important in the ecological recovery and restoration of degraded and contaminated soils. Selection of the most appropriate plant species is a crucial step to guarantee the success of phytoremediation processes. Forage crops (alfalfa) and fast-growing trees (poplars) have been previously used for phytoremediation of contaminated soils (Lingua et al., 2008; Panchenko et al., 2017), in many cases following a monoculture approach. However, nowadays agroforestry intercropping strategies are attracting much interest because of their potential environmental, economic, and social benefits (Hotelier-Rous et al., 2020). In particular, poplar-alfalfa intercropping has high potential for *in-situ* phytoremediation of contaminated soils, since it combines the deep rooting system of poplar trees with the extensive colonization of the soil ecosystem by alfalfa roots, where nitrogen fixation takes place, contributing to lower maintenance (fertilization requirements) of the intercropping system and to enhancement of contaminant degradation. Another important issue is the presence and growth of spontaneous vegetation during the phytoremediation process. The potential interference of spontaneous vegetation with the growth of the planted vegetation (here, alfalfa and/or poplar), as well as potential synergistic or antagonistic effects regarding the impact of plants on soil physicochemical and biological properties, need to be carefully assessed. Our results indicated that the alfalfa crop limited colonization by spontaneous vegetation (Figure 1). Certain traits of alfalfa, such as its high growth rate, profuse root system, and nitrogen-fixation ability, can explain the capacity of this leguminous species to limit the growth of or even to outcompete other herbaceous species. On the other hand, the poplar plots showed high levels of plant diversity; interestingly, the number of plant species and the total plant biomass increased with time, the latter reaching levels as high as 10 t DW ha^–1^. Among the spontaneous vegetation, *M. albus* appeared as a dominant pioneer species, showing the highest biomass productivity (12.5 t DW ha^–1^) among all the plant species. This leguminous species can compete effectively with alfalfa (see Pa treatment in Figure 1). The ability of *M. albus* to improve the health of degraded and contaminated soils (Halecki and Klatka, 2018), especially if associated with mycorrhizal fungi (Hernández-Ortega et al., 2012) or endophytic bacteria (Mitter et al., 2017, 2020), without requiring high levels of nutrients (low fertilization requirements) makes it a good candidate for long-term phytoremediation programs.

The growth of alfalfa when intercropped with poplar (Figures 2A,C) not only reduced the biomass and diversity of spontaneous vegetation but also decreased the woody biomass of poplar trees in the first growing season (Pa treatment, Figure 2B). The competition between the two crops for space and resources (e.g., water and nutrients) in the critical post-planting period can explain this observation. Interestingly, mycorrhizal inoculation of poplar trees counteracted the negative effect caused by alfalfa on woody biomass (Pia treatment, Figure 2B). The positive contribution of mycorrhizal fungi to plant establishment and growth in disturbed and stressed ecosystems is well known (Gould et al., 1996). According to Quoreshi and Khasa (2008), inoculation of seedlings or cuttings of poplars with mycorrhizal fungi ensures their optimal field performance. Under our experimental conditions, inoculation of poplar cuttings improved the establishment of young trees in the intercropping system (Figure 2B). The commercial mycorrhizal inoculum used here contains *Rhizopogon* spp., *Scleroderma* spp., and *Pisolithus tinctorius* (ectomycorrhizae), and *Glomus intraradices* and *Glomus mosseae* (arbuscular endomycorrhizae). These ectomycorrhizae are known to increase poplar biomass (Cripps, 2001; Gunderson et al., 2007; Ma et al., 2008). Analogous results have been reported with poplar trees growing in contaminated soils when inoculated with arbuscular endomycorrhizae belonging to Glomeromycota (Quoreshi and Khasa, 2008; Cicatelli et al., 2010; Pallara et al., 2013; Zhang H. et al., 2019). Ectomycorrhizal and endomycorrhizal fungi can improve plant quality and performance because they (i) may alleviate the (oxidative) stress induced by metals (Godbold et al., 1998) and organic contaminants (Małachowska-Jutsz and Kalka, 2010), and (ii) facilitate soil exploration by roots for water and nutrient acquisition (Marschner and Dell, 1994; Giovannetti et al., 2001). The enhancement of stress tolerance in poplars derived from mycorrhizal inoculation is a key beneficial effect for phytoremediation purposes.

The above-mentioned differences among treatments in the first growing season (July 2017) disappeared in the second growing season (July 2018), indicating that once they are well established, poplar trees can effectively compete with the surrounding vegetation. In this regard, certain acclimation traits were observed during the more stressful period of the first growing season. Leaves are plastic organs that can vary greatly in morphology, anatomy, and physiology in response to environmental conditions. The increase in the sclerophylly of leaves, as reflected in the reduction of SLA values observed in all treatments in July 2017 (Figure 2A), is associated with enhanced water-use efficiency under stress (soil water stress) and is a common strategy of phenotypic adjustment in plants (Wellstein et al., 2017). Accordingly, in the second growing season (July 2018), when the environmental conditions were milder and the trees had become established, all poplar treatments showed a larger total leaf area per tree (Figure 2C). In 2017, in all poplar treatments, the transplant stress affected the values of the plant parameters determined here. This transplant stress was confirmed by the higher levels of the de-epoxidation index (Figure 2C) and tocopherol content (Figure 3C) in the poplar leaves. De-epoxidation of violaxanthin to antheraxanthin and then to zeaxanthin, as well as increased concentrations of antioxidants such as tocopherols in leaves, are photoprotective mechanisms observed in species of the genus *Populus* under stress conditions (Niinemets et al., 2003; Jia et al., 2020).

### Soil Microbial Properties

The lower levels of soil microbial activity, biomass, and bacterial functional diversity observed in the first growing season (July 2017) could be at least partly due to the combined effect of the removal of vegetation cover and tilling just before the beginning of the experiment, in order to prepare the site for the field experiment. Microbial soil properties have proved to be very sensitive to tilling practices (Kabiri et al., 2016). This early period was also critical for vegetation performance (Figures 2, 3) and plant diversity (Figure 1). In any case, this was a transient effect, as in the second growing season (July 2018), once the vegetation was well established and the soil structure had consolidated, the levels of these microbial parameters increased. Changes in soil environmental conditions are well known to influence microbial growth, activity, and diversity (Kabiri et al., 2016; Steinauer et al., 2016). Importantly, root exudates from the poplars, alfalfa, and spontaneous vegetation most likely contributed to this enhanced soil microbial activity and biomass. Successful phyto- and bio-remediation of contaminated soils requires acclimation by soil microorganisms (Abdollahinejad et al., 2020).

Soil enzyme activities control nutrient cycling and have often been used as bioindicators of soil health (Epelde et al., 2009; Gómez-Sagasti et al., 2012). In general, the soil enzyme activities showed no significant differences among treatments (Figure 6); only the Pa treatment resulted in significantly higher levels. Intercropping significantly (*p* < 0.05) increased OEA values compared to the monoculture plots, regardless of whether or not the poplar trees were inoculated with mycorrhizae (Figure 6). Intercropping of herbaceous and woody plant species has been reported to enhance soil enzyme activity compared to monoculture systems (Cui et al., 2018; Zeng et al., 2019). This enhancement is often attributed to an increase in the amount and diversity of root exudates, which provide not only energy but also a wide variety of easily available substrates for extracellular enzymes (Allison and Vitousek, 2005; Steinauer et al., 2016). Considering that all plots were initially amended with compost, and therefore one would expect higher soil microbial activity at the beginning of the experiment (induced by application of the organic amendment) followed by lower activity as time progressed (Keener et al., 2000), the observed stimulation of soil microbial communities caused by the established vegetation is a very interesting finding from the point of view of the targeted positive effect of plant treatments on soil microbial communities. Our results emphasize the need to consider both the plants and the soil microbial communities when designing and monitoring phytoremediation processes, in terms of their capacity to reduce contaminant concentrations and improve soil health.

The abundances of bacterial (Figure 5A) and fungal (Figure 5B) communities in soil samples, reflected in the gene-copy numbers, were increased by the presence of alfalfa and mycorrhizal poplars grown in the company of spontaneous vegetation. This stimulatory effect of soil microbial communities was not observed when analyzing the SIR data (Figure 4B) or community-level physiological profiles with Biolog EcoPlates^TM^ (Figure 4C), where no differences between treatments were observed. This could be explained by the changes in microbial structural biodiversity induced by alfalfa growth and mycorrhizal inoculation, as observed in the levels of abundance of specific bacterial and fungal taxa (Figures 7, 8) and the lower values of α-diversity (Table 3). Root exudates (Steinauer et al., 2016) and mycorrhization (Asmelash et al., 2016) can affect the structural and functional diversity of soil microbial communities. The high functional redundancy of soil microbial communities (Girvan et al., 2005), as well as changes in the rhizosphere microbiome may explain the observed maintenance of microbial growth and activity.

At the beginning of the experiment, sowing alfalfa and transplanting poplar trees altered the composition of the existing native soil microbiota and initiated the development of a distinct soil microbial community. Mycorrhizal inoculation markedly affected the composition of the soil prokaryotic and fungal communities (Figure 9 and Supplementary Table 1). Alfalfa, on the other hand, increased the relative importance of certain abundant bacterial orders. Several dominant prokaryotic and fungal orders varied significantly, depending on the presence of alfalfa (Figure 7) and the use of the mycorrhizal inoculum (Figure 8). The phylum Proteobacteria dominated the prokaryotic rhizosphere communities of mycorrhizal poplar trees. Members of the orders Xanthomonadales (Gammaproteobacteria) and Rhizobiales (Alphaproteobacteria) were more abundant in soils where mycorrhizal poplars were planted, compared to soils with non-mycorrhizal poplars (Supplementary Figure 3). Members of the order Micrococcales (phylum Actinobacteria) were also abundant in the rhizosphere of poplar trees. These orders are likely to form part of the core rhizosphere microbiome of poplars, in both contaminated (Foulon et al., 2016) and non-contaminated soils (Shakya et al., 2013). Several members of these bacterial orders possess multifunctional plant growth-promoting traits, and therefore can promote plant growth *directly* by, for example, fixing nitrogen, solubilizing phosphorus and potassium from a limited nutrient pool, and producing phytohormones and siderophores; or *indirectly* by producing ACC deaminase activity, antibiotics, antifungal compounds, etc. (Hayat et al., 2010; Yadav et al., 2018). The existence of plant growth-promoting bacteria in their rhizosphere facilitates the establishment and growth of poplar trees in degraded and/or contaminated sites. Additionally, some unidentified orders of Ascomycota were detected in high abundance in soils where alfalfa and mycorrhizal poplars were present. Some studies have recognized the key role of the phylum Ascomycota in environments contaminated with organics (Aranda, 2016) and metals (Gil-Martínez et al., 2021). However, the role of Ascomycota in the transformation of soil contaminants remains poorly understood. Interestingly, members of Ascomycota have been found to be essential drivers of OM decomposition in soils where hybrid poplars are planted (Zheng et al., 2017). We can therefore conclude that our mycorrhizal inoculum enhanced the abundance of Proteobacteria, Actinobacteria, and Ascomycota, which in turn benefited not only plant growth but also soil OM mineralization and nutrient cycling, thus improving the fertility of the soil studied here.

Our study supports the existence of an intimate relationship between above-ground plant diversity and below-ground microbial diversity, as reported for other contaminated soils (Burges et al., 2017). Soils colonized by spontaneous vegetation showed not only the highest plant diversity (Figures 1, 9) but also the highest richness of soil bacteria and fungi (Table 3). Some treatments, such as alfalfa sowing (Figure 7) and mycorrhizal inoculation (Figure 8), reduced plant and soil microbial diversity but favored the abundance of specific microbial taxa. These changes in soil microbial composition did not affect the overall soil microbial activity, probably owing to the above-mentioned high functional redundancy of soil microbial communities.

An important conclusion of the present study is that, although the concentration of organic contaminants was not reduced in this 2-year experiment by any of the phytoremediation treatments, soil health (reflected in the levels of microbial biomass, activity, and diversity parameters) was effectively improved. Our findings broaden current knowledge of the positive effects of alfalfa sowing on soil microbial biomass, and the benefits of intercropping for the stimulation of enzyme activity in soils contaminated with organic compounds. Finally, *Melilotus albus* appears to be a suitable candidate for phytoremediation of abandoned vacant lands contaminated with organic compounds.

## Data Availability Statement

The datasets presented in this study can be found in online repositories. The names of the repository/repositories and accession number(s) can be found below: European Nucleotide Archive [accession: http://www.ebi.ac.uk/ena/data/view/PRJEB44358].

## Author Contributions

JB and CG conceived and designed the study. MG-S, CG, IA, and JB had major roles in writing the manuscript. MG-S, JU, FM, UA, and AH were actively involved in field and laboratory work, data analysis, and interpretation. JV, JB, and CG were responsible for acquiring funding. All authors meet all established criteria for inclusion in the authorship and have approved the submitted version.

## Conflict of Interest

The authors declare that the research was conducted in the absence of any commercial or financial relationships that could be construed as a potential conflict of interest.

## Footnotes

1 www.biopoplar.com
