# Supplementary material for: Mycorrhizal-Assisted Phytoremediation and Intercropping Strategies Improved the Health of Contaminated Soil in a Peri-Urban Area
Source: Front Plant Sci. 2021 Jul 2;12:693044. doi: 10.3389/fpls.2021.693044 (PMC8283827; doi:10.3389/fpls.2021.693044)
Supplement: Supplementary file 1 [file Data_Sheet_1.docx]

Supplementary Material

## Supplementary Figures


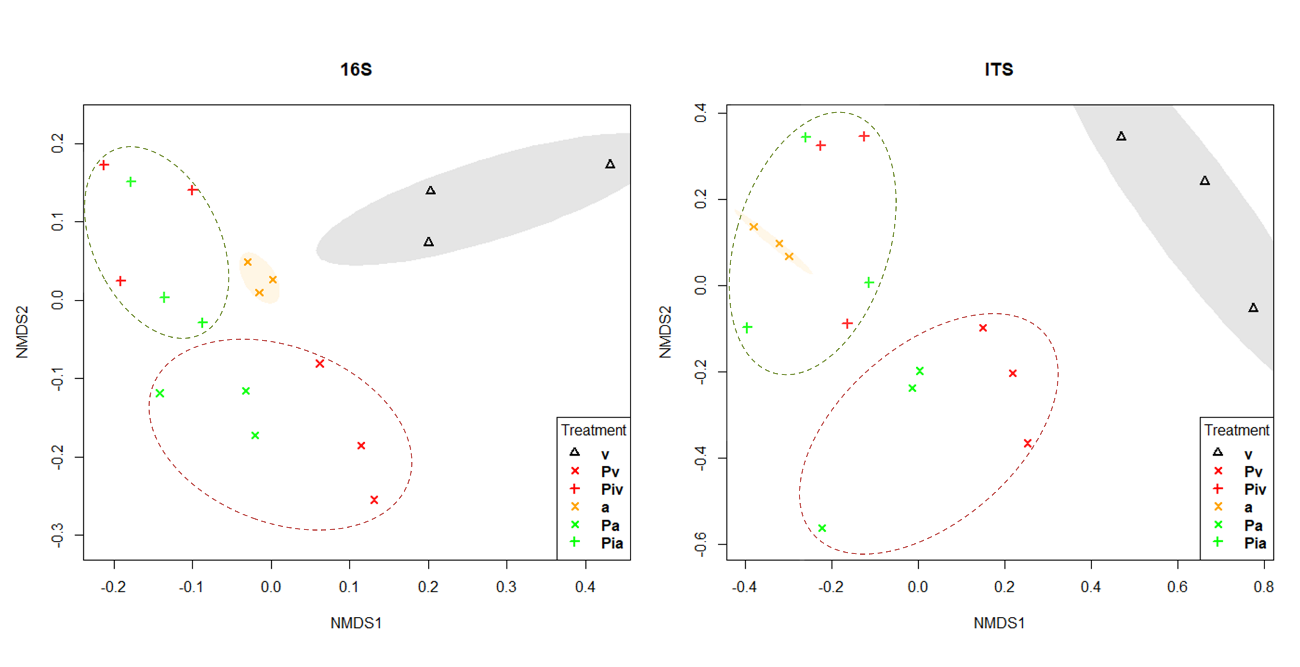


## Supplementary Figure 1. Non-metric multidimensional scaling (NMDS) analysis representing patterns of soil (A) prokaryotic and (B) eukaryotic OTU composition at the end of the experiment (2018, tf). Bray-Curtis dissimilarities of community composition based on relative OTU abundances from 16S and ITS rRNA amplicons are represented as distance in the diagram. Samples are labeled according to legend were: a: planting of alfalfa; Pa: intercropping of non-mycorrhizal poplars with alfalfa; Pia: intercropping of mycorrhizal poplars with alfalfa; Pv: planting of non-mycorrhizal poplars with spontaneous vegetation; Piv: planting of mycorrhizal poplars with spontaneous vegetation; v: spontaneous vegetation (unplanted control).


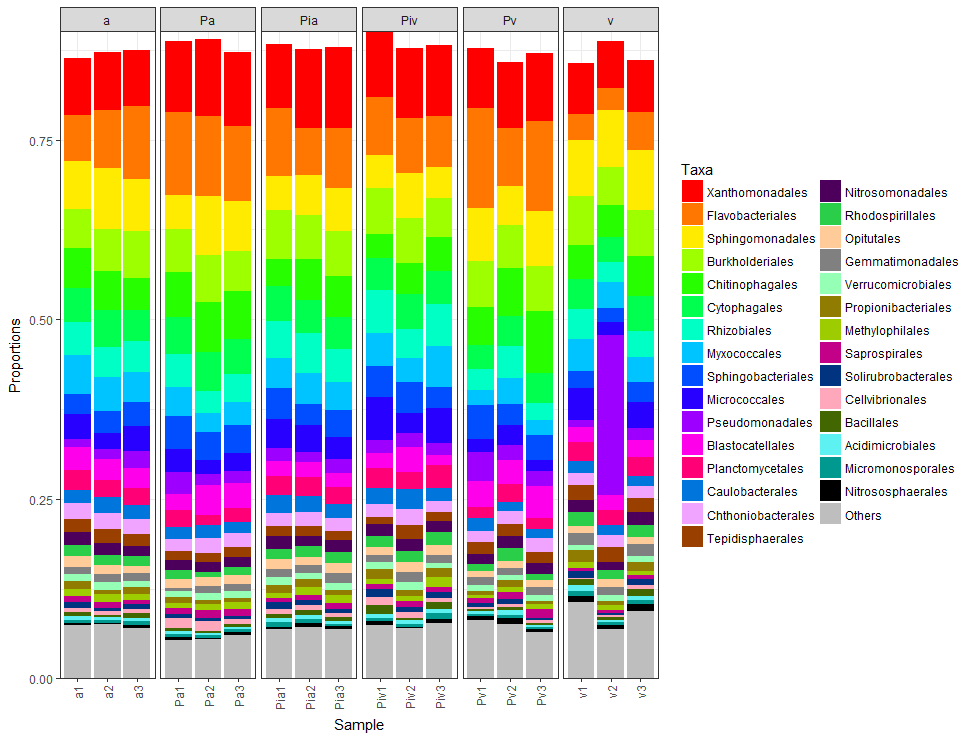


## Supplementary Figure 2. Barplot representing the distribution of the 30 most abundant prokaryotic taxa (at order rank) for each individual soil sample at the end of the experiment (2018, tf). Treatments were as follows: a: planting of alfalfa; Pa: intercropping of non-mycorrhizal poplars with alfalfa; Pia: intercropping of mycorrhizal poplars with alfalfa; Pv: planting of non-mycorrhizal poplars with spontaneous vegetation; Piv: planting of mycorrhizal poplars with spontaneous vegetation; v: spontaneous vegetation (unplanted control).


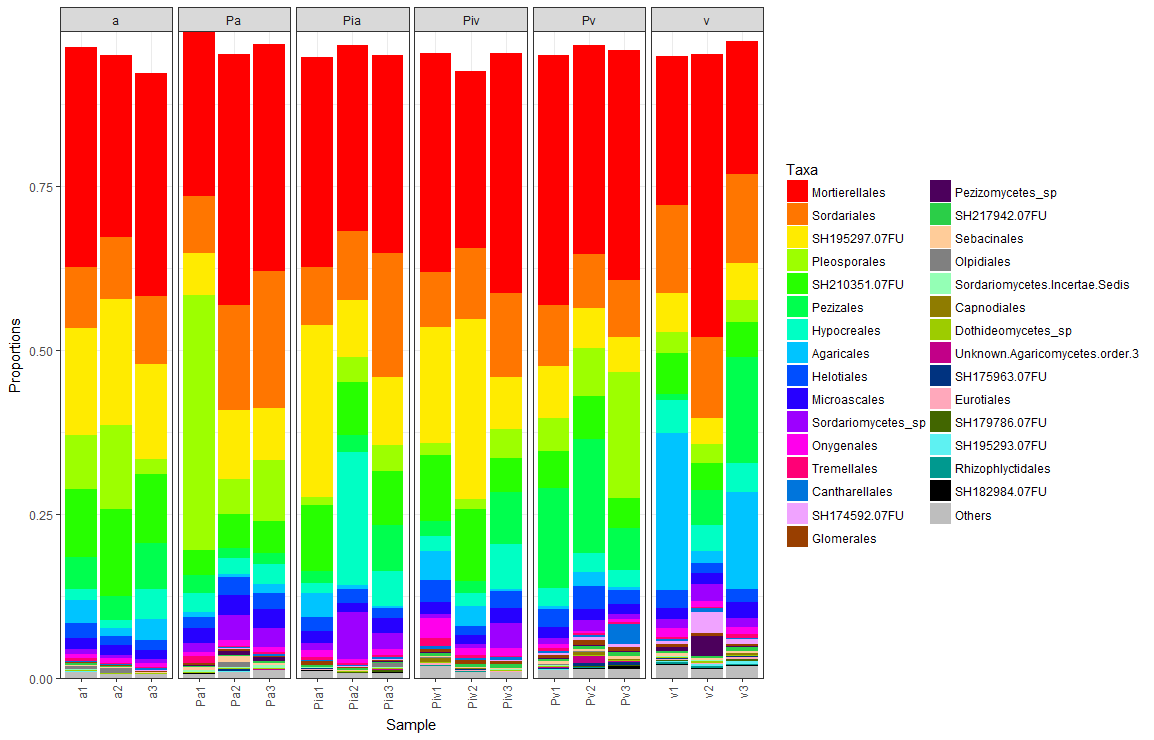


## Supplementary Figure 3. Barplot representing the distribution of the 30 most abundant fungal taxa (at order rank) for each individual soil sample at the end of the experiment (2018, tf). Treatments were as follows: a: planting of alfalfa; Pa: intercropping of non-mycorrhizal poplars with alfalfa; Pia: intercropping of mycorrhizal poplars with alfalfa; Pv: planting of non-mycorrhizal poplars with spontaneous vegetation; Piv: planting of mycorrhizal poplars with spontaneous vegetation; v: spontaneous vegetation (unplanted control).

## Supplementary Tables

## Supplementary Table 1. Relative abundances of soil prokaryotic and fungal orders at the end of the experiment (2018). Effect of the experimental factors “alfalfa planting” (A) and “mycorrhizal inoculation” (B). Only those taxa representing at least a 0.1% relative abundance for the whole prokaryotic and fungal communities are shown. Differences are based on pooled variances *t*-test or Welch's *t*-test.

| **(A) Experimental factor “alfalfa planting”** | | | |
| --- | --- | --- | --- |
| **Prokaryotic orders (%)** | | | |
| **Taxon** | **Alfalfa** | **Non-alfalfa** | ***p* value** |
| Cytophagales | 4.8 ± 0.3 | 4.2 ± 0.5 | 0.012 |
| Opitutales | 1.3 ± 0.2 | 1.1 ± 0.2 | 0.029 |
| Nitrososphaerales | 0.33 ± 0.07 | 0.59 ± 0.22 | 0.003 |
| Oceanospirillales | 0.40 ± 0.07 | 0.31 ± 0.07 | 0.008 |
| KD4-96 | 0.26 ± 0.07 | 0.43 ± 0.16 | 0.011 |
| Desulfurellales | 0.16 ± 0.05 | 0.23 ± 0.07 | 0.015 |
| Gaiellales | 0.13 ± 0.04 | 0.21 ± 0.09 | 0.019 |
| Clostridiales | 0.07 ± 0.03 | 0.16 ± 0.10 | 0.015 |
| Anaerolineales | 0.06 ± 0.02 | 0.15 ± 0.08 | 0.005 |
| **Fungal orders (%)** | | | |
| **Taxon** | **Alfalfa** | **Non-alfalfa** | ***p* value** |
| Glomerales | 0.20 ± 0.18 | 0.50 ± 0.12 | 0.001 |
| SH217942.07FU (Unknown Ascomycota) | 0.20 ± 0.09 | 0.47 ± 0.13 | <0.001 |
| Olpidiales | 0.29 ± 0.31 | 0.05 ± 0.06 | 0.036 |
| Capnodiales | 0.03 ± 0.03 | 0.19 ± 0.21 | 0.043 |
| Dothideomycetes_sp | 0.06 ± 0.03 | 0.15 ± 0.07 | 0.004 |
| **(B) Experimental factor “mycorrhizal inoculation”** | | | |
| **Prokaryotic orders (%)** | | | |
| **Taxon** | **Inoculated** | **Non-inoculated** | ***p* value** |
| Xanthomonadales | 10.0 ± 1.0 | 8.6 ± 1.3 | 0.036 |
| Sphingomonadales | 5.3 ± 0.8 | 7.2 ± 1.2 | 0.002 |
| Chitinophagales | 4.6 ± 0.9 | 5.9 ± 1.2 | 0.037 |
| Rhizobiales | 5.2 ± 0.7 | 3.8 ± 0.8 | 0.001 |
| Micrococcales | 4.1 ± 1.2 | 2.7 ± 0.9 | 0.018 |
| Blastocatellales | 2.2 ± 0.7 | 3.1 ± 0.8 | 0.026 |
| Planctomycetales | 2.7 ± 0.3 | 2.2 ± 0.5 | 0.038 |
| Caulobacterales | 2.3 ± 0.4 | 1.7 ± 0.3 | 0.002 |
| Tepidisphaerales | 1.3 ± 0.3 | 1.7 ± 0.3 | 0.031 |
| Solirubrobacterales | 0.84 ± 0.21 | 0.60 ± 0.21 | 0.033 |
| Acidobacteria group 7b | 0.13 ± 0.03 | 0.28 ± 0.13 | 0.014 |
| Desulfurellales | 0.15 ± 0.03 | 0.22 ± 0.06 | 0.014 |
| Rickettsiales | 0.17 ± 0.02 | 0.12 ± 0.03 | 0.008 |
| Chlorobiales | 0.10 ± 0.02 | 0.15 ± 0.03 | <0.001 |
| Legionellales | 0.14 ± 0.04 | 0.10 ± 0.02 | 0.005 |
|  | | | |
|  | | | |
| *(Continued)* | | | |
| **(B) Experimental factor “mycorrhizal inoculation”** | | | |
| **Fungal orders (%)** | | | |
| **Taxon** | **Inoculated** | **Non-inoculated** | ***p* value** |
| SH195297.07FU (Unknown Ascomycota) | 16.4 ± 8.8 | 9.1 ± 4.9 | 0.039 |
| Onygenales | 1.3 ± 0.8 | 0.7 ± 0.3 | 0.032 |
| Sebacinales | 0.09 ± 0.03 | 0.36 ± 0.21 | 0.008 |
